# Short-interval variability and test-retest reliability of Alzheimer’s disease plasma biomarkers in a clinically heterogenous cohort

**DOI:** 10.1186/s13195-026-02180-8

**Published:** 2026-10-27

**Authors:** Millie Beament, Katharine Wiltshire, Christopher R S Belder, Emily Abel, Melanie Hart, Amanda J Heslegrave, Michael P Lunn, Miguel Rosa-Grilo, Owen Swann, Jonathan M Schott, Henrik Zetterberg, Nick C Fox, Ashvini Keshavan

**Affiliations:** 1 Dementia Research Centre, Department of Neurodegenerative Disease, UCL Queen Square Institute of Neurology, University College London, London, WC1N 3AR UK; 2 UK Dementia Research Institute at UCL, University College London, London, UK; 3 Neurology Department, Royal Adelaide Hospital and Queen Elizabeth Hospital, Adelaide, Australia; 4 School of Medicine, Adelaide University, Adelaide, Australia; 5 Neuroimmunology & CSF Laboratory, National Hospital for Neurology and Neurosurgery, Queen Square, London, UK; 6 Department of Neuroinflammation, UCL Queen Square Institute of Neurology, London, UK; 7 MRC Centre for Neuromuscular Diseases, UCL Queen Square Institute of Neurology, London, UK; 8 Department of Psychiatry and Neurochemistry, Institute of Neuroscience and Physiology, the Sahlgrenska Academy at the University of Gothenburg, Mölndal, Sweden; 9 Clinical Neurochemistry Laboratory, Sahlgrenska University Hospital, Mölndal, Sweden; 10 Department of Pathology and Laboratory Medicine, University of Wisconsin School of Medicine and Public Health, Madison, WI USA; 11 Wisconsin Alzheimer’s Disease Research Center, University of Wisconsin School of Medicine and Public Health, University of Wisconsin-Madison, Madison, WI USA; 12 Centre for Brain Research, Indian Institute of Science, Bangalore, India

**Keywords:** Blood-based biomarkers, Alzheimer’s disease, Test-retest reliability, Phosphorylated tau-217

## Abstract

**Background:**

Assessment of short-term within-subject variability and test-retest reliability of Alzheimer’s disease (AD)-related plasma biomarkers is important for real-world clinical implementation and interpretation in research studies. In this study we aimed to assess short-term variability and reliability of core AD plasma biomarkers, proposed ratios, and evaluate a broader proteomic panel.

**Methods:**

Two serial plasma samples were collected from each of the 92 participants enrolled in observational research studies over intervals ranging from 30 minutes to three months. Test-retest reliability and within-subject variability were assessed for 131 biomarkers measured on three immunoassays (Lumipulse G plasma p-tau217; Fujirebio, NULISAseq CNS Disease Panel and NULISAqpcr AD 5-Plex; Alamar Biosciences). We subsequently evaluated the stability of a dual cut-point approach for AD probability diagnostic categorisation and examined whether within-subject variability was associated with inter-sample interval (time between serial blood sampling).

**Results:**

All core AD plasma biomarkers assessed in this study (Aβ42, BD-p-tau181, BD-p-tau217, BD-p-tau231, p-tau181, p-tau217, p-tau231, GFAP and NfL) demonstrated strong correlations (r ≥ 0.79) and high reliability (ICC ≥ 0.84). Within-subject variability was moderate to low for core AD plasma biomarkers. Using a validated dual cut-point approach for plasma p-tau217, most individuals (92%) remained within the same diagnostic category (high/indeterminate/low) across both timepoints, and no individual moved across two boundaries (i.e. from high to low AD probability categorisation or vice versa). For the majority of analytes, within-subject variability was not associated with inter-sample interval after adjustment for age and sex, however, the core AD plasma biomarker, NfL did have a significant association.

**Conclusions:**

While core AD plasma biomarkers had limited variability and high reliability over short-intervals, other central nervous system (CNS)-relevant biomarkers showed greater variability, which may be relevant for interpretation in association studies and clinical applications.

**Supplementary Information:**

The online version contains supplementary material available at https://doi.org/10.1186/s13195-026-02180-8.

## Background

Blood-based biomarkers (BBMs) are accessible, non-invasive and cost-effective tools for assessing Alzheimer’s disease (AD) pathology [1]. BBMs are increasingly relevant to diagnosis and prognosis of AD and will be increasingly important for determining eligibility for disease-modifying therapies (DMTs) [2]. As such, this study investigates short-term within-subject variability and test-retest reliability of core AD plasma biomarkers [3], alongside a broader panel of central nervous system (CNS)-relevant biomarkers, to distinguish clinically relevant changes in biomarker concentrations from expected variability.

BBMs capturing aspects of AD pathology include plasma amyloid beta (Aβ40 and Aβ42) [4], phosphorylated tau (p-tau181, p-tau217 and, p-tau231) [5], neurofilament light chain (NfL) and glial fibrillary acidic protein (GFAP) [6, 7]. Among these, plasma p-tau217 has emerged as the leading candidate diagnostic BBM for AD [7], with studies demonstrating strong concordance with beta-amyloid pathology as assessed by cerebrospinal fluid (CSF) or amyloid positron emission tomography (PET) in cognitively impaired individuals [8–11]. Newer assays which specifically measure brain derived (BD) tau isoforms mitigate the influence of peripherally derived tau on plasma measurements, thereby improving specificity [12]. Further, for some BBMs, a ratio of two markers (Aβ42/Aβ40 and p-tau217/Aβ42) has been suggested to offer improved performance [13–15].

These biomarkers capture only part of the complex pathophysiology and heterogeneity of AD. Updated frameworks emphasise the importance of additional markers reflecting inflammation, vascular pathology and synucleinopathy [3]. Current markers of vascular pathology are limited to infarctions or white matter hyperintensities on structural imaging, however, emerging fluid biomarkers include, vascular cell adhesion molecule-1 (VCAM-1) and vascular endothelial growth factor (VEGF) [16]. Neuroinflammatory markers include those associated with activated microglia and astrocytes, such as GFAP and triggering receptor expressed on myeloid cells 2 (TREM2) [17]. Moreover, synaptic dysfunction is increasingly recognised as an early feature of AD, with studies showing that synaptosomal-associated protein 25 (SNAP-25) and related synaptic markers correlate with both cognitive decline and established measures of synaptic injury [18].

These observations highlight the value of high-plex BBM platforms capable of simultaneously quantifying multiple analytes with high sensitivity and specificity [19]. NULISA (NUcleic acid-Linked Immuno-Sandwich Assay), a recently developed high-plex proteomic platform based on proximity extension assay technology demonstrates background suppression and attomolar sensitivity. This enables the simultaneous measurement of multiple CNS-relevant analytes across a broad dynamic range in plasma [20].

Successful implementation of BBMs requires an understanding of factors that may influence measurements. Many pre-analytical factors have been investigated previously [21–23] with guidelines to ensure stable biomarker concentrations across assay platforms [24]. Clinical confounders such as body mass index (BMI) and chronic kidney disease have also been shown to affect levels of BBMs [25, 26]. Further, a small number of studies suggest time of day and food intake possibly contribute to variability [27–29]. By contrast, few studies have examined reliability and variability of BBMs over short-intervals on single and low-plex immunoassays [30–33] and no studies to-date have evaluated high-plex platforms.

We therefore aimed to assess the short-term within-subject variability and test–retest reliability of plasma biomarkers measured using single-, low- and high-plex immunoassays using two serial plasma samples collected from the same individual over time intervals ranging from 30 minutes to three months in cognitively impaired and unimpaired participants. Secondary objectives included examining the stability of a plasma p-tau217 dual cut-point approach [11] for AD probability diagnostic categorisation, assessing the effect of inter-sample interval (time between serial blood sampling) on within-subject variability and comparison of BBMs across assays.

## Methods

### Study population

Recruitment for this prospective test-retest reliability study took place between October 2018 and July 2025. Participants from research studies at the Dementia Research Centre, University College London (UCL), which recruit individuals with and without cognitive impairment, were included if they had at least 2 serial blood sample collections, as part of their study enrolment, taken a maximum of 3 months apart. All participants provided written informed consent prior to sample collection. Ethical approval was granted by the NHS Health Research Authority (REC references: Biomarkers & Rapid Imaging for Dementia Diagnosis; 21/LO/0815, Biomarkers and Genetics in Cognitive Disorders; 03/N049, Longitudinal Study of familial Alzheimer’s Disease; 11/LO/0753, Cerebrospinal fluid collection in patients with suspected neurodegenerative diseases; 12/LO/1504 and Healthy control biofluid study: CONFluid; 19/LO/0913). All studies were performed in accordance with the 1964 Declaration of Helsinki and its later amendments.

### Sample collection and processing

Serial blood sampling was performed on 92 participants either on the same day or within 92 days (~ 3 months). Participants were not asked to fast prior to sample collection. Blood was collected by peripheral venepuncture into 8 mL K_2_-ethylene-diaminetetraacetic acid (EDTA)-plasma tubes according to standardised procedures. Samples were processed and centrifuged at 2,000 g for 10 min at room temperature within 2 h of collection. Plasma was aliquoted and stored in 2 mL polypropylene screw top cryovials at – 80 °C until analysis.

### Biomarker assays

#### Lumipulse G plasma p-tau217

Paired plasma samples were tested in singlicate at the Neuroimmunology and CSF Laboratory, University College London Hospital (UCLH), on the Lumipulse G1200 fully automated immunoassay platform, using commercially available kits for Lumipulse G plasma p-tau217, a subsequently CE-marked chemiluminescent enzyme immunoassay (Fujirebio, Lot# 5082 and 5102), according to the manufacturer’s recommendation. Prior to analysis, samples were thawed at room temperature, briefly vortexed and centrifuged at 2,000 g for 10 min. All plasma sample pairs were tested within the same run and were quantifiable above the lower limit of quantification (LLOQ) of 0.03 pg/mL, which was provided by the manufacturer and verified at this laboratory.

#### NULISAseq CNS disease panel

Paired plasma samples were tested in singlicate using the NULISAseq CNS Disease Panel (Alamar Biosciences, Lot# 2512001b3 and 2519607b8) at the Fluid Biomarker Laboratory, UCL, according to manufacturer’s instructions. Following thawing at room temperature, samples were centrifuged at 10,000 g for 10 min and 20 µl of each sample was transferred to the 96-well sample plate provided with the reagent kit. The plate was sealed and spun for 1 min at 1000 g. The automated Alamar Argo HT workflow was used. Briefly, immunocomplexes were formed with DNA-barcoded capture and detection antibodies which were subsequently captured on paramagnetic oligo-dT beads. Following washing the immunocomplexes were released and recaptured on streptavidin beads, before a final wash step, where the captured immunocomplexes DNA strand was ligated to generate a DNA reporter molecule, containing target and sample specific barcodes which were pooled into a single library for next generation sequencing [20]. The library was sequenced using the Illumina P2 100 cycle flow cell (Illumina part# 20100987, Reagent lot# 20975663, 20971773 and 21016296, Flow cell Lot# 20964548 and 21011698) on a NextSeq 1000. Sequencing data was processed using the Argo Command Centre (Alamar Biosciences) for normalisation and rescaled (add 1 and log2 transformed), to produce the output of NULISA protein quantification (NPQ) units, for each biomarker. All plasma sample pairs were run on the same plate on the version 3 NULISAseq CNS Disease Panel which detects 131 neuro-specific and inflammatory proteins.

#### NULISAqpcr AD 5-Plex

Only 80 paired plasma samples from the total cohort were available for testing in singlicate using the NULISAqpcr (quantitative polymerase chain reaction) AD 5-Plex assay (Alamar Biosciences, Lot# 2532501), which was due to kit availability. This assay measures; BD-p-tau217, Aβ42, NfL, GFAP and apolipoprotein (APOE) ε4, at the Fluid Biomarker Laboratory, UCL according to manufacturer’s instructions. Methodology is as in NULISAseq CNS disease panel, above; however, the DNA reporter molecules were instead amplified and quantified using target specific probes by qPCR. Quantification was determined from cycle threshold (Cq) values measured during the exponential phase of amplification, with values transformed to a linear scale using 2ˆ(37 − Cq) and calibrated using standard curves fitted with a 4-parameter logistic (4-PL) model. All plasma sample pairs were run on the same plate.

### Statistical analysis

All statistical analysis was performed in R, version 4.4.2. Age was summarised as a continuous variable using the median and interquartile range (IQR). The binary variable of sex, and the categorical variable of diagnosis, were presented as total numbers and percentages. Differences in age across diagnostic groups were assessed using the Kruskal–Wallis test, followed by pairwise Wilcoxon rank-sum tests with Benjamini–Hochberg adjustment for multiple comparisons. Differences in sex across groups were assessed using Fisher’s exact test. 

Correlations between paired measurements were assessed using Spearman rho and confidence intervals (CI) at 95% were determined with 2000 bootstraps resampling. Test-retest reliability was evaluated using intraclass correlation coefficient (ICC), calculated with a two-way random-effects model for absolute agreement and single measurements (ICC [2,1]) [34]. Higher values of Spearman rho and ICC indicate greater test–retest reliability. Within-subject variability was assessed using within-subject coefficient of variation (CV_w_), where for each participant, the standard deviation of the repeated measurements was calculated and divided by the participant-specific mean and expressed as a percentage. Lower CV_w_ values indicate lower within-subject variability. For p-tau217 measured on the Lumipulse G, and for BD-p-tau217, Aβ42, NfL and GFAP measured using the NULISAqpcr AD 5-Plex assay, Spearman rho, ICC and CV_w_ were calculated for both absolute concentrations and log_2_-transformed values. NULISAseq CNS Disease Panel biomarker results are reported in NPQ units on a log-transformed relative scale with Spearman rho and ICC calculated using the reported NPQ values. For CV_w_, NPQ units were back-transformed (2^NPQ^-1) to a linear scale, as suggested by the manufacturer, before being calculated. Where ratios were calculated using log₂-transformed values, the following convention was applied: log₂(Aβ42/Aβ40) was calculated as log₂(Aβ42) − log₂(Aβ40). 

Correlations between biomarkers measured on different assays were assessed using Spearman rho and CI at 95% were determined with 2000 bootstraps resampling. Finally, a linear regression model was used to evaluate whether longer inter-sample intervals were associated with greater relative CV_w_, adjusting for age and sex (CV_w_ ~ inter-sample interval + age + sex). To control for multiple testing across biomarkers, p values were adjusted using Benjamini–Hochberg false discovery rate (FDR), with adjusted *p*< 0.05 considered statistically significant.

## Results

### Participants

The 92 participants included had a median age of 52 years (IQR 39–64); 42 (46%) were female (Table 1). The cohort comprised 22 individuals with a clinical diagnosis of AD, 8 with other neurodegenerative disease diagnoses, 27 asymptomatic autosomal dominant Alzheimer’s disease (ADAD) asymptomatic mutation carriers (AMC), and 35 controls. Pairwise comparisons identified, ADAD AMC as significantly younger than the AD and other neurodegenerative disease groups (Wilcoxon rank sum tests p-value > 0.001), as expected. The proportion of males and females did not differ between groups (Fishers exact test *p*-value = 0.096) (Supplementary Table 1). (Fishers exact test *p*-value = 0.096) (Supplementary Table 1). The median inter-sampling interval was 85 min (range: 30 min to 92 days).

**Table 1:** Participant demographics and clinical characteristics

Characteristic	Value, *n* = 92
Age, years*	52, IQR 39–64
Female, n (%)	42 (45.7)
Clinical diagnosis, n (%)	
AD	23 (25)
Other neurodegenerative disease**	7 (7.6)
ADAD AMC	27 (29.3)
Controls	35 (38.0)

*Age is reported as the median, IQR = interquartile range

**Other neurodegenerative disease diagnoses included; 1 Cerebral amyloid angiopathy, 1 Vascular cognitive impairment, 5 Frontotemporal lobar degeneration

### Test-retest reliability of core AD plasma biomarkers measured on Lumipulse G and NULISAqpcr AD 5-Plex assays

We assessed test-retest reliability and within-subject variability of core AD biomarkers measured using the Lumipulse G plasma p-tau217 (Fujirebio) and the NULISAqpcr AD 5-Plex immunoassays (Alamar Biosciences). Performance was evaluated using absolute concentrations and log_2_-transformed values, with reliability assessed by Spearman rho and ICC and variability assessed by CV_w_ (Supplementary Table 2).

**Fig. 1:**
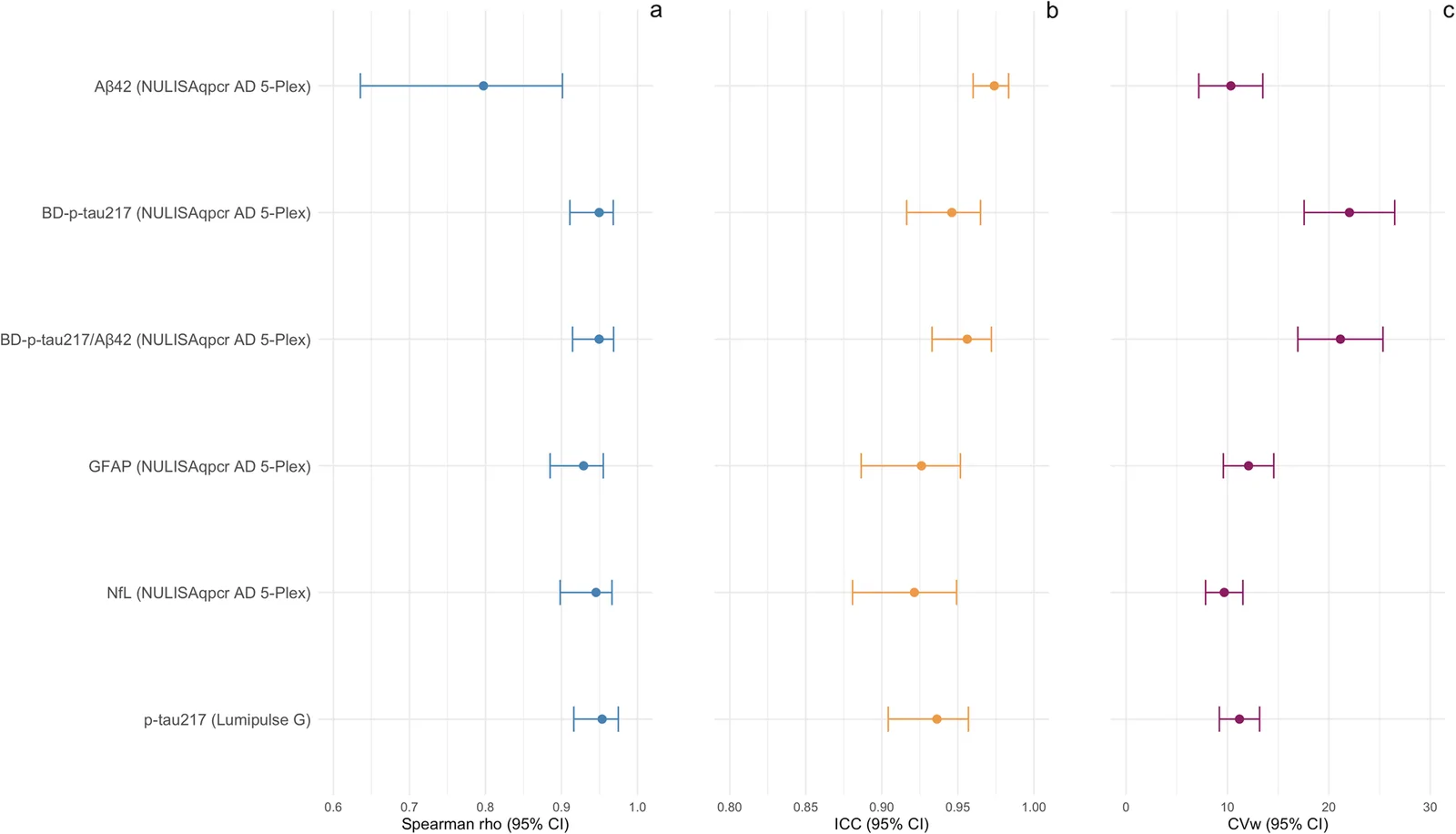
Test–retest reliability of core Alzheimer’s disease plasma biomarkers measured using the Lumipulse G (Fujirebio),  *N* = 92, and NULISAqPCR (quantitative polymerase chain reaction) AD 5-Plex (Alamar Biosciences),  *N* = 80, immunoassays. For each biomarker, test–retest reliability was assessed using (**a**) Spearman rho, (**b**) intraclass correlation coefficient (ICC), and (**c**) within-subject coefficient of variation (CV_w_). Points denote estimated values and error bars represent corresponding 95% confidence intervals (CIs), Spearman rho and ICC both have lower bounds of 0 and upper bounds of 1

All core AD biomarkers (Aβ42, BD-p-tau217, GFAP, NfL and p-tau217) demonstrated high reliability across repeated measures (Spearman rho ≥ 0.80 and ICC ≥ 0.92). Within-subject variability ranged from 9.7% (95% CI 7.8–11.5%) for NfL to 22.0% (95% CI 17.6–26.5%) for BD-p-tau217 (Fig. 1).

The BD-p-tau217/Aβ42 ratio did not improve reliability relative to BD-p-tau217 alone (Spearman rho 0.95) and resulted in only a modest reduction in within-subject variability (BD-p-tau217; CV_w_ 22.0%, 95% CI 17.6–26.5% vs. BD-p-tau217/Aβ42; 21.1%, 95% CI 16.9–25.3%).

The reliability of p-tau217 measured using the Lumipulse G assay (Spearman rho 0.95, 95% CI 0.92–0.97, ICC 0.94, 95% CI 0.90–0.96) was comparable to that of BD-p-tau217 measured using the NULISAqpcr AD 5-Plex assay (Spearman rho 0.95, 95% CI 0.91–0.97, ICC 0.95, 95% CI 0.92–0.96). However, within-subject variability was lower on the Lumipulse platform (p-tau217; CV_w_ 11.2%, 95% CI 9.2–13.2%, BD-ptau217; CV_w_ 22.0%, 95% CI 17.6–26.5%).

We assessed whether reliability metrics and within-subject variability differed across clinical diagnostic groups. A difference was considered significant based on non-overlapping 95% CI (Supplementary Table 3). There were no significant group differences for Spearman rho across the core AD biomarkers. There was a significant difference in ICC between the AD and control groups for plasma p-tau217 measured using Lumipulse G. For plasma Aβ42 measured using the NULISAqpcr AD 5-plex assay, significant differences in ICC were observed between the AD and ADAD AMC groups and between the ADAD AMC and control groups. For plasma NfL and GFAP also measured using the NULISAqpcr AD 5-plex assay, significant differences in ICC were observed between the AD and control groups and between the ADAD AMC and control groups. Finally, there was a significant difference in CV_w_ between the AD and control groups for the plasma BD-p-tau217/Aβ42 ratio measured using the NULISAqpcr AD 5-plex assay.

### Test-retest reliability of plasma biomarkers measured on NULISAseq CNS disease panel

We also assessed test-retest reliability and within-subject variability on the NULISAseq CNS Disease Panel, a high-plex proteomic assay that measures 131 CNS-relevant analytes. APOE ε 4 was excluded from whole-platform comparisons, as recommended by the manufacturer, because it is reported as a binary variable rather than a continuous measurement on the NULISAseq platform. Corresponding data are provided in Supplementary Table 4. In addition, Aβ42/40 was the only analyte ratio evaluated on the NULISAseq platform.

**Fig. 2:**
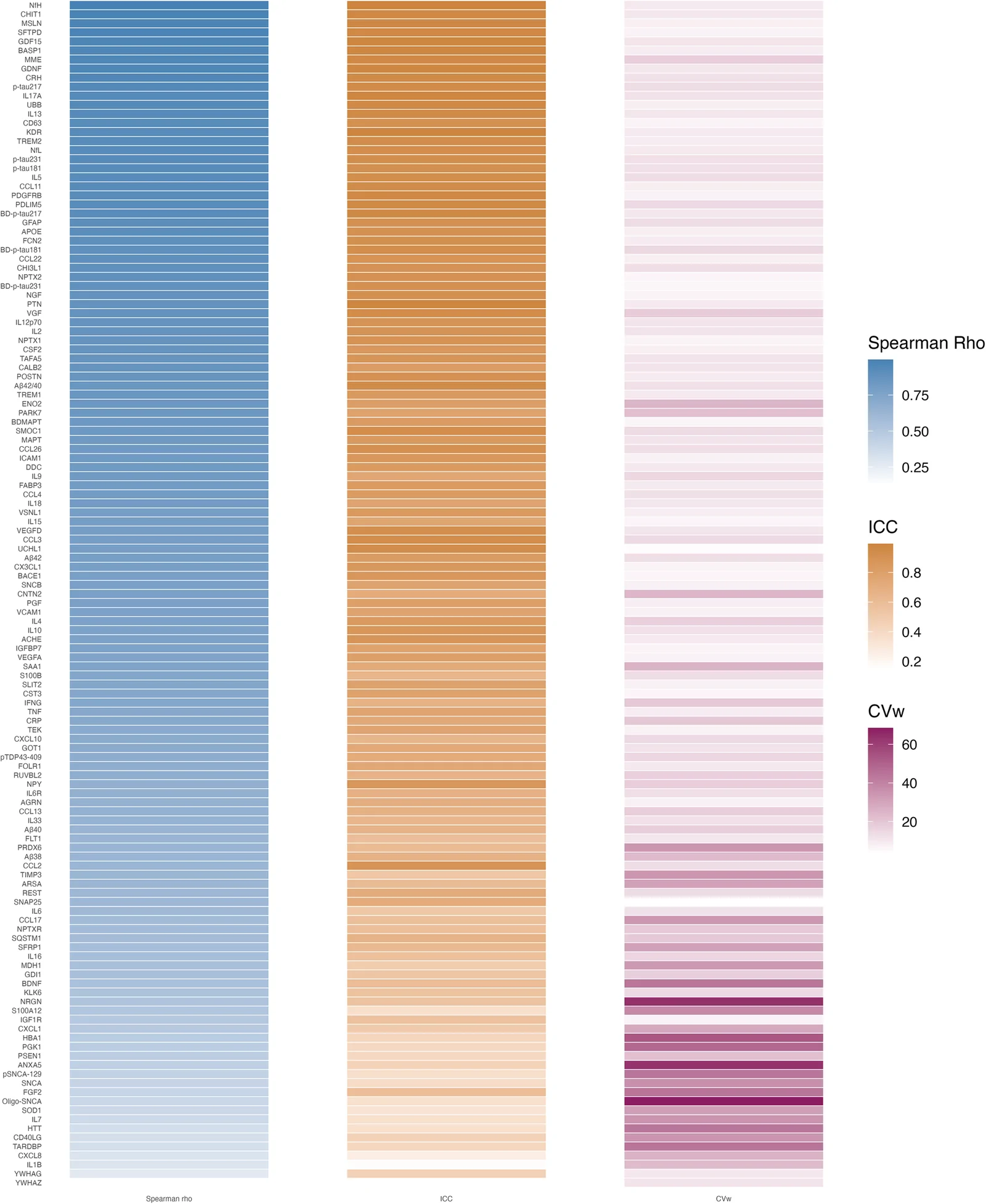
Ranking of test-retest reliability metrics for plasma biomarkers quantified using the NULISAseq CNS Disease Panel (Alamar Biosciences),  *N* = 92. Biomarkers were ranked according to their performance for each reliability metric. Darker colours for Spearman rho and intraclass correlation coefficient (ICC) indicate higher correlations and test-retest reliability, with lighter colour for within-subject coefficient of variation (CV_w_) indicating lower within-subject variability. Spearman rho and ICC both have lower bounds of 0 and upper bounds of 1

Based on predefined study thresholds, strong correlations (Spearman rho ≥ 0.75) were observed for 74 analytes, moderate correlations (Spearman rho = 0.46–0.74) for 43 analytes, and low correlations (Spearman rho ≤ 0.45) for 14 analytes. Similarly high test-retest reliability (ICC ≥ 0.75) was observed for 78 analytes, moderate reliability (ICC = 0.46–0.74) for 37 analytes, and low reliability (ICC ≤ 0.45) for 16 analytes. The majority, 101 out of 132, analytes had a CV_w_ of less than 20% (Fig. 2).

The Aβ42/40 ratio demonstrated higher reliability than Aβ42 (Aβ42/40; Spearman rho 0.85, 95% CI 0.76–0.91, ICC 0.95, 95% CI 0.93–0.97, Aβ42; Spearman rho 0.79, 95% CI 0.62–0.90, ICC 0.84, 95% CI 0.76–0.89), within-subject variability was similar (CV_w_ Aβ42/40 12.5%, 95% CI 8.2–16.8%, CV_w_ Aβ42 13.0%, 95% CI 9.6–16.3%) (Supplementary Table 4).

### Cross-assay correlation of plasma BD-p-tau217 and p-tau217

Strong concordance across assays was observed for both plasma p-tau217 and BD-p-tau217. P-tau217, as quantified on the Lumipulse G and the NULISAseq CNS Disease Panel, were strongly correlated (Spearman rho = 0.88, *P* < 0.001, Supplementary Fig. 2a). Similarly, BD-p-tau217 measurements from the NULISAseq CNS Disease Panel and NULISAqpcr AD 5-Plex assay showed high cross-assay correlation (Spearman rho = 0.90,  *p* < 0.001, Supplementary Fig. 2b).

### AD probability categorisation stability across timepoints

**Fig. 3:**
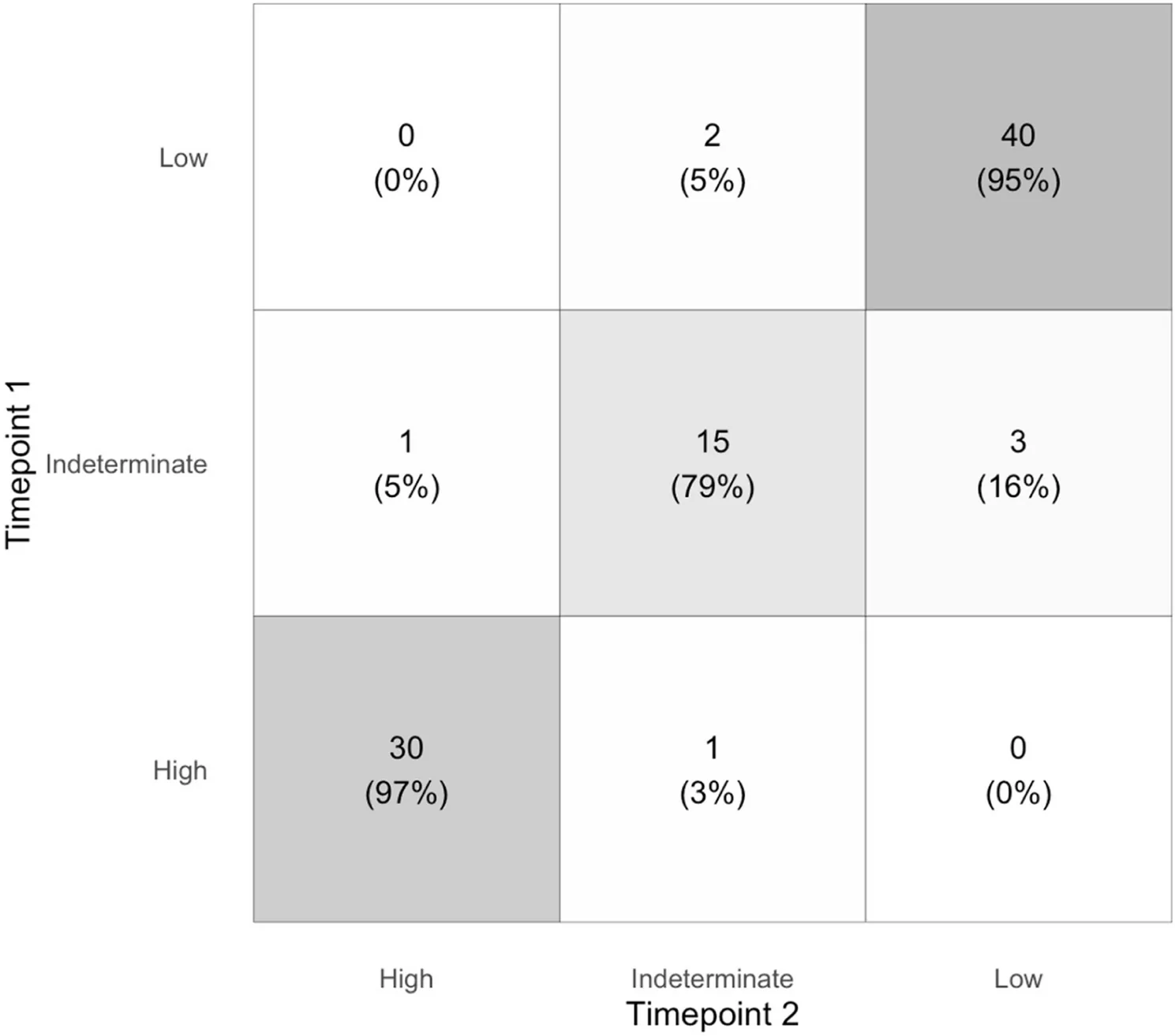
AD probability categorisation stability across timepoints as assessed by Lumipulse G p-tau217 assay (Fujirebio). Cut-points used were: >0.422 pg/mL = high, 0.153–0.422 pg/mL = indeterminate, and < 0.153 pg/mL = low

We classified p-tau217 concentrations measured on the Lumipulse G at each timepoint according to previously defined thresholds derived from an independent cohort (> 0.422 pg/mL = high, 0.153–0.422 pg/mL = indeterminate, and < 0.153 pg/mL = low) [11], and determined the proportion of individuals reclassified across the low, indeterminate, and high AD probability categories. Most individuals remained within the same category at both timepoints, with 8% (7/92, 1 other neurodegenerative disease, 4 ADAD AMC and 2 controls) crossing a single cut-point boundary (Fig. 3). Averaging p-tau217 values (arithmetic mean) across the two timepoints did not reduce the proportion of individuals in the indeterminate category.

### Effect of longer inter-sample intervals on within-subject variability

**Fig. 4:**
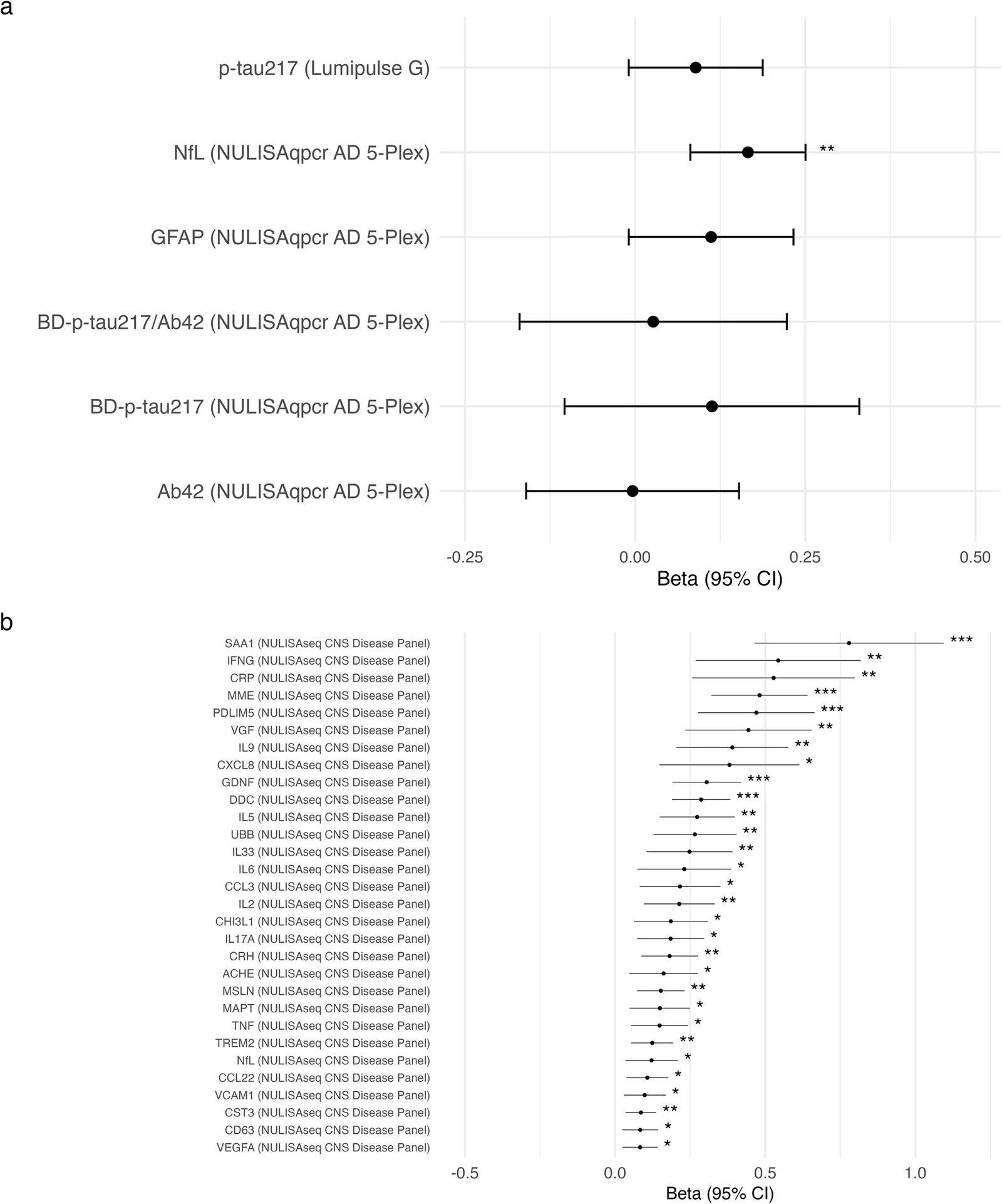
Effect of inter-sample interval on within-subject variability for plasma biomarkers measured using the (**a**) Lumipulse G (Fujirebio) and NULISAqpcr AD 5-plex (Alamar Biosciences) immunoassays and (**b**) all significant plasma biomarkers measured using the NULISAseq CNS Disease Panel. Each point represents an individual biomarker effect estimate (beta) and error bars represent the corresponding 95% confidence intervals (CIs). Adjusted p-values are NS unless other indicated; *p<0.05, **p < 0.01, ***p<0.001

Linear regression models assessed whether longer inter-sample intervals were associated with greater CV_w_, adjusting for age and sex (Supplementary Table 5). Following FDR correction, 30 biomarkers measured on the NULISAseq CNS Disease Panel (Fig. 4b), and 1 biomarker measured on the NULISApqcr AD 5-Plex assay (Fig. 4a) showed significant associations with longer time between sampling, this included the core AD biomarker, NfL.

## Discussion

This study reports the short-term within-subject variability and test-retest reliability of plasma biomarkers measured using single-, low- and high-plex immunoassays. To our knowledge, this is the first study to evaluate a high-plex immunoassay in this context; previous work has focused on single- and low-plex platforms [30–33], none of which included the Lumipulse G plasma p-tau217 assay (Fujirebio) or the NULISAqpcr AD 5-plex assay (Alamar, Biosciences). Across all assays, core AD plasma biomarkers demonstrated strong correlations and excellent reliability. Within-subject variability was within expected bounds for the absolute and relative quantification of the respective assays. Most analytes measured on the NULISAseq CNS Disease Panel demonstrated strong to moderate correlations, high to moderate test-retest reliability, and moderate to low within-subject variability. Several showed less stable performance.

Core AD plasma biomarkers measured over short-intervals, during which no clinical progression would be expected, demonstrated excellent test-retest reliability, with high Spearman rho and ICC values and low within-subject variability. Ratio-based measures showed variable performance. Reliability was improved for Aβ42/Aβ40 compared to Aβ42 alone when measured using the NULISAseq CNS Disease Panel. In contrast BD-p-tau217/Aβ42 measured on the NULISAqpcr AD 5-Plex assay did not show improved reliability compared to BD-p-tau217 alone. Previous work has reported reduced variability when using the p-tau217/BD-tau ratio compared with p-tau217 [32], and several studies suggest that ratios can improve diagnostic accuracy for detecting pathology [13–15]. Notably, the first FDA-approved plasma biomarker test incorporates a p-tau217/Aβ42 ratio. However, our findings add nuance to this evidence. Ratios did not consistently enhance test-retest reliability and given the known impact of preanalytical variability on several analytes, including Aβ42 [22, 24, 35], their diagnostic implementation should therefore be guided by clear evidence of added clinical value. Finally, differences between clinical diagnostic groups were observed for several of the core AD biomarkers. Any interpretation of the effect of clinical diagnosis on the reliability and within-subject variability of these biomarkers should be limited due to small group numbers.

The within-subject variability observed for the assays providing absolute quantification (Lumipulse G p-tau217 assay and NULISAqpcr AD 5-Plex assay) was consistent with previous reports of single and low-plex immunoassays. Cullen et al. reported test-retest variability of 20.0% for p-tau217, here we report a CV_w_ of 11.2% [33]. The relative quantification provided by the NULISAseq CNS Disease Panel, limits direct cross-platform comparisons, however, for the majority of the core AD biomarkers, within-subject variability was comparable. BD-p-tau217 showed increased within-subject variability on the NULISAqpcr AD 5-plex assay compared to the highplex platform. This likely reflects, at least in part, the reporting of NPQ units on a relative scale rather than as absolute concentrations. Further cross-platform analyses demonstrated strong agreement between assays, with high correlations for BD-p-tau217 (Spearman rho = 0.90) and p-tau217 (Spearman rho = 0.88). These findings are consistent with previous reports of cross-platform concordance, which have ranged from 0.78 to 0.94 [6, 36, 37].

We next evaluated the stability of a dual cut-point approach using paired plasma samples collected over short-intervals. Applying cut-points (> 0.422 pg/mL = high, 0.153–0.422 pg/mL = indeterminate, and < 0.153 pg/mL = low) derived from stage 1 of The Alzheimer’s Disease Diagnosis and Plasma Phospho-Tau217 study [11], we found that only 8% of individuals crossed a single cut-point boundary, none of which had a clinical diagnosis of AD. These findings have implications for clinical interpretation, particularly for individuals with values near diagnostic thresholds, where even small changes may alter classification. In such cases, results should be interpreted within the broader clinical context. This also raises the question of whether short-interval repeat testing may be beneficial for individuals close to cut-points to improve diagnostic confidence. We observed an average absolute percentage difference of 15.8% between the repeated measurements and a CV_w_ of 11.2%, providing an estimate of short-interval variability of plasma p-tau217 on the Lumipulse platform in our cohort. Values exceeding this range may indicate additional pre-analytical influences.

We further assessed whether averaging paired p-tau217 values reduced the proportion of individuals classified within the indeterminate, ‘grey zone’. A dual cut-point approach typically classifies approximately 20% of individuals to this category, for whom additional confirmatory testing (e.g. CSF or PET) is recommended. Reducing the proportion of individuals within the ‘grey zone’ would facilitate the clinical implementation of BBM testing [38, 39]. However, averaging paired plasma p-tau217 measurements in this study did not reduce the number of individuals classified within the ‘grey zone’. This contrasts with structural imaging studies, where repeated imaging reduces variance and within-subject variability [40, 41]. Collectively, these findings suggest that short-interval repeated blood sampling provides limited benefit for interpreting the plasma p-tau217 immunoassay (Lumipulse, Fujirebio) when applying these cut-points.

When examining the effect of sampling intervals, we identified 30 plasma biomarkers measured on the NULISAseq CNS Disease Panel and one plasma biomarker on the NULISAqpcr AD 5-Plex assay that showed modestly increased within-subject variability over longer inter-sample intervals, including NfL; this was reflective of a gradual increase in within-subject variability overtime and not driven by outliers at the longer time intervals. This result is potentially reflecting sensitivity to physiological changes. NfL is a dynamic biomarker that tracks neurodegeneration over time [42, 43], and some increases in variability, even over relatively short intervals, is therefore expected. Moreover, this study included a wide range of inter-sample intervals (30 min to 92 days, median 85 min), with sample pairs collected on the same day potentially reflecting very short-term aspects of biological variability, such as time of day, food intake and physiological factors, rather than chronic biological variability seen over weeks to months, which could be suggestive of health changes and other exposures. Further studies assessing both aspects of biological variability are needed to understand these findings.

In addition to core AD biomarkers, we assessed test-retest reliability of 121 other CNS-relevant biomarkers measured on the NULISAseq CNS Disease Panel. Both oligomeric and monomeric alpha-synuclein exhibited low correlation and reduced reliability, while oligomeric alpha-synuclein also demonstrated moderate within-subject variability. This is notable given prior reports of differential expression in individuals with mild cognitive impairment compared with controls [44]. Similarly, TAR DNA-binding protein (TARDBP), which has been associated with AD [45], showed low correlation and reduced reliability in this study. Further, as highlighted in Supplementary Fig. 1 when the NULISAseq CNS Disease Panel analytes are grouped by ontology (as defined by manufacturer), ‘synuclein and synaptic disorder’ related analytes appear to show lower correlation and reduced reliability and higher within-subject variability compared to other groups. These findings highlight the importance of evaluating both within-subject variability and reliability for interpretation of plasma biomarkers, particularly in association studies.

This study has several limitations. The interval between repeat sampling was relatively broad (30 min to 92 days). Although we limited inclusion to samples collected within three months, during which substantial neurodegenerative changes are unlikely to occur, we observed increased variability over longer sampling intervals for some biomarkers, which may reflect underlying physiological changes and warrants further investigation. Furthermore, participants were younger in age than a typical memory clinic demographic, with relatively few individuals with symptomatic neurodegenerative disease. This may lead to underestimation of variability seen in older patients with symptomatic neurodegenerative disease and other co-pathologies which might influence measured biomarker levels even over short intervals. Participants were not required to fast, and although most were likely non-fasted, the impact of fasting status was not formally assessed. In addition, we did not account for factors such as BMI or chronic kidney disease, although these are unlikely to have changed substantially over the sampling intervals studied.

Finally, NULISAseq data are reported as NPQ values on a log-transformed relative scale. In contrast, the NULISAqpcr AD 5-Plex and Lumipulse G p-tau217 assays provide absolute concentrations. Calculation of CV_w_ on log_2_-transformed absolute concentration data yielded values that were not directly comparable with conventional clinically reported CV_w_ estimates. To facilitate comparison with clinical laboratory performance metrics for these clinically relevant assays, we presented analyses using the raw concentration data with log_2_-transformed data available in supplementary (Supplementary Table 2). NULISAseq NPQ values were first back-transformed before CV_w_ was calculated to account for their logarithmic scale.

## Conclusion

In conclusion, core AD biomarkers show strong correlations and high test-retest reliability, with low within-subject variability across assay platforms. This study suggests caution when interpreting associations of biomarkers with disease where their short-term variability is not fully understood. Further studies should repeat this work in larger, biomarker defined, cohorts where other factors impacting variation in physiological states, such as fasting and sample timing, are accounted for.

## Supplementary Information

Supplementary Material 1.

## Data Availability

The datasets generated and/or analysed during the current study are not publicly available due to protection of confidentiality of participants but are available from the corresponding author on reasonable request.

## Abbreviations

Aβ: Amyloid beta
AD: Alzheimer’s disease
ADAD: Autosomal dominant Alzheimer’s disease
APOE: Apolipoprotein
BBM: Blood-based biomarkers
BMI: Body mass index
BD: Brain derived
CI: Confidence interval
CNS: Central nervous system
CSF: Cerebrospinal fluid
CV_w_: Within-subject coefficient of variation
Cq: Cycle threshold
DMT: Disease modifying therapy
EDTA: Ethylene-diaminetetraacetic acid
FDR: False discovery rate
GFAP: Glial fibrillary acidic protein
LLOQ: Lower limit of quantification
ICC: Intraclass correlation coefficient
IQR: Interquartile range
NfL: Neurofilament light chain
NPQ: NULISA protein quantification
NULISA: NUcleic acid-Linked Immuno-Sandwich Assay
P-tau: Phosphorylated tau
PET: Positron emission tomography
qPCR: Quantitative polyermase chain reaction
SNAP25: Synaptosomal-associated protein 25
TARDBP: TAR DNA-binding protein
TREM2: Triggering receptor expressed on myeloid cells 2
UCL: University College London
UCLH: University College London Hospital
4-PL: 4-parameter logistic
