# Supplementary material for: Short-interval variability and test-retest reliability of Alzheimer’s disease plasma biomarkers in a clinically heterogenous cohort
Source: Alzheimers Res Ther. 2026 Oct 27;18:238. doi: 10.1186/s13195-026-02180-8 (PMC13711881; doi:10.1186/s13195-026-02180-8)
Supplement: Supplementary file 1 [file 13195_2026_2180_MOESM1_ESM.docx]

Table of Contents

[Supplementary Table 1. Participant demographics and clinical characteristics separated by clinical diagnosis	2](#_Toc236215723)

[Supplementary Table 2. Test–retest reliability of core Alzheimer’s disease plasma biomarkers	2](#_Toc236215724)

[Supplementary Table 3. Test–retest reliability of core Alzheimer’s disease plasma biomarkers separated by disease category	3](#_Toc236215725)

[Supplementary Table 4: Test-retest reliability of plasma biomarkers quantified by the NULISAseq CNS disease panel	3](#_Toc236215726)

[Supplementary Table 5. Association between inter-sample interval and within-subject variability of plasma biomarkers	7](#_Toc236215727)

[Supplementary Table 6. Plasma biomarkers measured using the NULISAseq CNS Disease Panel, with full names and manufacturer-defined CNS panel categories (Alamar Biosciences)	12](#_Toc236215728)

[Supplementary Figure 1: Test-retest reliability metrics for plasma biomarkers quantified using the NULISAseq CNS Disease Panel (Alamar Biosciences) ordered by manufacturer defined panel category.	16](#_Toc236215729)

[Supplementary Figure 2. Cross-assay correlation of plasma p-tau217 and BD-p-tau217	17](#_Toc236215730)

[Supplementary Figure 3. Plasma biomarkers, measured on the NULISAseq CNS Disease Panel, association between inter-sample interval and within-subject variability (CVw) following false discovery rate (FDR) correction.	19](#_Toc236215731)

## **Supplementary Table 1. Participant demographics and clinical characteristics separated by clinical diagnosis**

|  | AD (N=23) | Other ND (N=7) | ADAD AMC (N=27) | Controls (N=35) | p-value |
| --- | --- | --- | --- | --- | --- |
| Age, years* | 61,  IQR 57–68 | 70,  IQR 62–74 | 39,  IQR 34–45 | 52,  IQR 34–63 | >0.001 (1) |
| Female, n (%) | 10 (43.5) | 1 (14.3) | 17 (63.0) | 14 (40.0) | 0.097 (2) |

*Age is reported as the median.

(1) Kruskal-Wallis test.

(2) Fishers exact test.

Abbreviations: ND, Neurodegenerative disease, AMC, asymptomatic mutation carrier, IQR, interquartile range.

## **Supplementary Table 2. Test–retest reliability of core Alzheimer’s disease plasma biomarkers**

| Biomarker | Spearman rho (95% CI) | ICC (95% CI) | CV_w_ (95% CI) |
| --- | --- | --- | --- |
| Lumipulse G | | | |
| p-tau217 | 0.95 (0.92–0.97) | 0.94 (0.90–0.96) | 11.2 (9.2–13.2) |
| Log_2_p-tau217 | 0.95 (0.91–0.97) | 0.98 (0.97–0.99) | 17.6 (-22.0–57.2) |
| NULISAqpcr AD 5-plex | | | |
| Aβ42 | 0.80 (0.64–0.90) | 0.97 (0.96–0.98) | 10.3 (7.2–13.5) |
| Log_2_Aβ42 | 0.80 (0.64–0.90) | 0.89 (0.84–0.93) | 3.2 (2.0–4.3) |
| BD-p-tau217 | 0.95 (0.91–0.97) | 0.95 (0.92–0.96) | 22.0 (17.6–26.5) |
| Log_2_BD-p-tau217 | 0.95 (0.91–0.97) | 0.94 (0.91–0.96) | 5.8 (-25.8–37.5) |
| BD-p-tau217/ Aβ42 | 0.95 (0.91–0.97) | 0.96 (0.93–0.97) | 21.1 (16.9–25.3) |
| Log_2_BD-p-tau217/ Aβ42 | 0.95 (0.91–0.97) | 0.96 (0.93–0.97) | -4.9 (-5.9– -3.8) |
| GFAP | 0.93 (0.88–0.95) | 0.93 (0.89–0.95) | 12.1 (9.6–14.6) |
| Log_2_GFAP | 0.93 (0.88–0.96) | 0.93 (0.89–0.96) | 5.1 (4.0–6.2) |
| NfL | 0.95 (0.90–0.97) | 0.92 (0.88–0.95) | 9.7 (7.8–11.5) |
| Log_2_NfL | 0.95 (0.90–0.97) | 0.94 (0.91–0.96) | 6.8 (5.3–8.2) |

Test–retest reliability was evaluated for core Alzheimer’s disease plasma biomarkers measured using the Lumipulse G (Fujirebio), N=92, and NULISA qPCR (quantitative polymerase chain reaction) AD 5-Plex (Alamar Biosciences), N=80, immunoassays using Spearman rho, intraclass correlation coefficient (ICC), and within-subject coefficient of variation (CV_w_). Values are presented with corresponding 95% confidence intervals (CIs), Spearman rho and ICC both have lower bounds of 0 and upper bounds of 1. Spearman rho p < 0.001 for all biomarkers.

## **Supplementary Table 3. Test–retest reliability of core Alzheimer’s disease plasma biomarkers separated by disease category**

| Biomarker | Disease category | N | Spearman Rho  (95% CI) | ICC  (95% CI) | CV_w_  (95% CI) |
| --- | --- | --- | --- | --- | --- |
| Lumipulse G | | | | | |
| p-tau217 | AD | 23 | 0.87 (0.65–0.96) | 0.89 (0.76–0.95) | 13.13 (8.30–17.95) |
|  | Controls | 35 | 0.82 (0.60–0.94) | 0.99 (0.97–0.99) | 8.72 (6.36–11.07) |
|  | Other ND | 7 | 0.86 (0.19–1.00) | 0.98 (0.87–1.00) | 8.45 (2.60–14.30) |
|  | ADAD AMC | 27 | 0.94 (0.82–0.98) | 0.89 (0.78–0.95) | 13.45 (8.99–17.90) |
| NULISAqpcr AD 5-plex | | | | | |
| BD-p-tau217 | AD | 22 | 0.92 (0.76–0.98) | 0.92 (0.81–0.96) | 15.15 (9.68–20.62) |
|  | Controls | 30 | 0.82 (0.64–0.90) | 0.82 (0.65–0.91) | 25.64 (16.51–34.76) |
|  | ADAD AMC | 27 | 0.89 (0.71–0.96) | 0.92 (0.83–0.96) | 23.63 (16.26–31.00) |
| Aβ42 | AD | 22 | 0.75 (0.40–0.94) | 0.80 (0.58–0.91) | 12.29 (6.81–17.77) |
|  | Controls | 31 | 0.61 (0.28–0.82) | 0.78 (0.59–0.89) | 6.69 (6.44–8.94) |
|  | ADAD AMC | 27 | 0.49 (0.04–0.86) | 0.97 (0.94–0.99) | 12.92 (4.83–21.02) |
| NfL | AD | 22 | 0.83 (0.53–0.94) | 0.83 (0.64–0.93) | 10.82 (6.52–15.11) |
|  | Controls | 31 | 0.95 (0.87–0.98) | 0.97 (0.95–0.99) | 8.10 (5.74–10.46) |
|  | ADAD AMC | 27 | 0.92 (0.80–0.97) | 0.76 (0.52–0.88) | 10.59 (7.04–14.13) |
| GFAP | AD | 22 | 0.81 (0.50–0.95) | 0.86 (0.68–0.94) | 14.24 (8.76–19.72) |
|  | Controls | 31 | 0.89 (0.74–0.97) | 0.99 (0.97–0.99) | 10.80 (6.78–14.83) |
|  | ADAD AMC | 27 | 0.93 (0.82–0.98) | 0.86 (0.71–0.93) | 11.80 (7.64–15.97) |
| BD-p- tau217/ Aβ42 | AD | 22 | 0.86 (0.61–0.98) | 0.94 (0.86–0.98) | 13.27 (8.78–17.77) |
|  | Controls | 30 | 0.80 (0.60–0.90) | 0.82 (0.65–0.91) | 26.86 (18.73–34.99) |
|  | ADAD AMC | 27 | 0.92 (0.78–0.98) | 0.84 (0.68–0.92) | 21.20 (13.78–28.63) |

Test–retest reliability was evaluated for core Alzheimer’s disease plasma biomarkers, across the four disease categories, measured using the Lumipulse G (Fujirebio), N=92, and NULISA qPCR (quantitative polymerase chain reaction) AD 5-Plex (Alamar Biosciences), N=80, immunoassays using Spearman rho, intraclass correlation coefficient (ICC), and within-subject coefficient of variation (CV_w_). Values are presented with corresponding 95% confidence intervals (CIs) Spearman rho and ICC both have lower bounds of 0 and upper bounds of 1. Spearman rho p < 0.001 for all biomarkers. Abbreviations: ND, Neurodegenerative disease, AMC, asymptomatic mutation carrier.

## **Supplementary Table 4: Test-retest reliability of plasma biomarkers quantified by the NULISAseq CNS disease panel**

| Biomarker | Spearman rho  (95% CI) | ICC  (95% CI) | CV_w_  (95% CI) |
| --- | --- | --- | --- |
| ACHE | 0.76 (0.62–0.87) | 0.88 (0.82–0.92) | 9.8 (7.5-12.1) |
| AGRN | 0.66 (0.50–0.77) | 0.70 (0.58–0.79) | 7.8 (6.6-9) |
| ANXA5 | 0.44 (0.24–0.61) | 0.44 (0.26–0.59) | 63.6 (54.7-72.4) |
| APOE | 0.91 (0.85–0.95) | 0.91 (0.87–0.94) | 8.4 (6.2-10.6) |
| APOE ε4 | 0.90 (0.81–0.95) | 1.00 (1.00–1.00) | 49.2 (35-63.3) |
| ARSA | 0.62 (0.44–0.76) | 0.61 (0.47–0.72) | 31.4 (25.4-37.4) |
| Aβ38 | 0.62 (0.45–0.76) | 0.67 (0.54–0.77) | 23.5 (18.9-28.1) |
| Aβ40 | 0.64 (0.49–0.75) | 0.67 (0.53–0.77) | 17.7 (14.3-21.1) |
| Aβ42 | 0.79 (0.62–0.90) | 0.84 (0.76–0.89) | 13.0 (9.6-16.3) |
| Aβ42/Aβ40 | 0.85 (0.76–0.91) | 0.95 (0.93–0.97) | 12.5 (8.2-16.8) |
| BACE1 | 0.78 (0.65–0.87) | 0.86 (0.80–0.91) | 6.6 (5.4-7.7) |
| BASP1 | 0.96 (0.91–0.99) | 0.99 (0.98–0.99) | 9.0 (6.6-11.4) |
| BDMAPT | 0.83 (0.73–0.89) | 0.84 (0.77–0.89) | 6.5 (5.3-7.6) |
| BDNF | 0.55 (0.34–0.71) | 0.59 (0.44–0.71) | 44.0 (37.8-50.2) |
| BD-p-tau181 | 0.90 (0.82–0.95) | 0.94 (0.91–0.96) | 14.8 (12.3-17.3) |
| BD-p-tau217 | 0.92 (0.86–0.95) | 0.96 (0.93–0.97) | 10.5 (8.6-12.4) |
| BD-p-tau231 | 0.89 (0.88–0.95) | 0.91 (0.87–0.94) | 6.1 (5-7.2) |
| CALB2 | 0.86 (0.79–0.91) | 0.83 (0.75–0.88) | 11.2 (9.2-13.3) |
| CCL11 | 0.93 (0.87–0.96) | 0.94 (0.92–0.96) | 8.5 (7.2-9.8) |
| CCL13 | 0.65 (0.49–0.79) | 0.68 (0.56–0.78) | 17.6 (14.1-21.2) |
| CCL17 | 0.59 (0.40–0.75) | 0.58 (0.43–0.70) | 34.1 (27.4-40.9) |
| CCL2 | 0.62 (0.46–0.75) | 0.89 (0.84–0.93) | 13.2 (10.7-15.8) |
| CCL22 | 0.90 (0.83–0.94) | 0.90 (0.85–0.93) | 8.1 (6.6-9.5) |
| CCL26 | 0.82 (0.72–0.89) | 0.92 (0.88–0.94) | 13.0 (10.7-15.3) |
| CCL3 | 0.80 (0.68–0.87) | 0.94 (0.90–0.96) | 14.1 (11.3-16.9) |
| CCL4 | 0.81 (0.67–0.91) | 0.83 (0.76–0.89) | 12.6 (10.1-15.1) |
| CD40LG | 0.34 (0.12–0.55) ** | 0.45 (0.28–0.60) | 34.8 (28.2-41.4) |
| CD63 | 0.93 (0.89–0.95) | 0.92 (0.88–0.94) | 7.1 (5.8-8.3) |
| CHI3L1 | 0.89 (0.82–0.94) | 0.90 (0.86–0.93) | 12.9 (10.4-15.4) |
| CHIT1 | 0.98 (0.97–0.99) | 0.97 (0.95–0.98) | 10.9 (6.4-15.3) |
| CNTN2 | 0.77 (0.67–0.85) | 0.72 (0.60–0.80) | 24.8 (20.1-29.5) |
| CRH | 0.96 (0.92–0.97) | 0.96 (0.94–0.97) | 12.3 (10.2-14.4) |
| CRP | 0.71 (0.52–0.86) | 0.73 (0.62–0.82) | 19.8 (13.7-25.9) |
| CSF2 | 0.87 (0.79–0.92) | 0.86 (0.80–0.91) | 8.2 (6.4-9.9) |
| CST3 | 0.73 (0.58–0.83) | 0.78 (0.69–0.85) | 6.3 (5.2-7.4) |
| CX3CL1 | 0.79 (0.65–0.88) | 0.86 (0.79–0.90) | 6.9 (5.7-8.1) |
| CXCL1 | 0.48 (0.29–0.67) | 0.50 (0.33–0.64) | 28.9 (23.2-34.5) |
| CXCL10 | 0.70 (0.50–0.86) | 0.66 (0.52–0.76) | 14.5 (10.4-18.7) |
| CXCL8 | 0.31 (0.07–0.53) ** | 0.26 (0.06–0.44) | 25.9 (21-30.7) |
| DDC | 0.82 (0.72–0.89) | 0.84 (0.76–0.89) | 10.2 (7.9-12.5) |
| ENO2 | 0.84 (0.77–0.88) | 0.82 (0.74–0.88) | 25.4 (20.9-29.9) |
| FABP3 | 0.81 (0.70–0.89) | 0.84 (0.76–0.89) | 9.6 (7.5-11.7) |
| FCN2 | 0.90 (0.84–0.94) | 0.92 (0.89–0.95) | 9.4 (8-10.9) |
| FGF2 | 0.41 (0.19–0.60) | 0.59 (0.44–0.71) | 44.6 (37-52.2) |
| FLT1 | 0.63 (0.48–0.75) | 0.60 (0.45–0.72) | 10.8 (9-12.6) |
| FOLR1 | 0.68 (0.54–0.78) | 0.74 (0.63–0.82) | 10.2 (8.7-11.7) |
| GDF15 | 0.97 (0.94–0.98) | 0.99 (0.98–0.99) | 11.1 (7.6-14.7) |
| GDI1 | 0.55 (0.36–0.70) | 0.53 (0.37–0.66) | 17.6 (14.2-20.9) |
| GDNF | 0.96 (0.91–0.98) | 0.98 (0.97–0.99) | 10.6 (8-13.1) |
| GFAP | 0.92 (0.87–0.94) | 0.92 (0.87–0.94) | 13.6 (11.5-15.8) |
| GOT1 | 0.70 (0.56–0.80) | 0.73 (0.62–0.81) | 11.5 (9.4-13.5) |
| HBA1 | 0.47 (0.28–0.64) | 0.44 (0.26–0.59) | 53.2 (45-61.4) |
| HTT | 0.34 (0.13–0.53) ** | 0.35 (0.16–0.52) | 44.2 (36.9-51.5) |
| ICAM1 | 0.82 (0.71–0.89) | 0.86 (0.80–0.91) | 7.7 (6.2-9.2) |
| IFNG | 0.72 (0.55–0.86) | 0.67 (0.54–0.77) | 19.5 (13.6-25.4) |
| IGF1R | 0.49 (0.30–0.65) | 0.57 (0.41–0.69) | 7.2 (5.9-8.4) |
| IGFBP7 | 0.76 (0.64–0.84) | 0.77 (0.68–0.84) | 6.9 (5.8-8.1) |
| IL10 | 0.77 (0.61–0.88) | 0.87 (0.81–0.91) | 12.3 (8.9-15.7) |
| IL12p70 | 0.87 (0.77–0.94) | 0.89 (0.84–0.92) | 11.2 (8.4-14.1) |
| IL13 | 0.94 (0.88–0.97) | 0.96 (0.94–0.97) | 10.5 (7.7-13.3) |
| IL15 | 0.80 (0.65–0.90) | 0.77 (0.66–0.84) | 6.7 (5.2-8.3) |
| IL16 | 0.57 (0.36–0.73) | 0.58 (0.43–0.70) | 15.3 (11.3-19.3) |
| IL17A | 0.94 (0.89–0.97) | 0.99 (0.98–0.99) | 11.8 (9.5-14.1) |
| IL18 | 0.81 (0.68–0.90) | 0.77 (0.67–0.84) | 10.8 (7.8-13.8) |
| IL1B | 0.30 (0.08–0.52) ** | 0.17 (-0.03–0.36) | 23.4 (18.2-28.6) |
| IL2 | 0.87 (0.78–0.93) | 0.89 (0.84–0.93) | 11.6 (9.1-14.1) |
| IL33 | 0.65 (0.49–0.77) | 0.65 (0.51–0.75) | 12.4 (9.5-15.4) |
| IL4 | 0.77 (0.65–0.85) | 0.85 (0.78–0.90) | 17.0 (14.1-20) |
| IL5 | 0.93 (0.87–0.96) | 0.93 (0.90–0.95) | 13.3 (10.4-16.3) |
| IL6 | 0.60 (0.39–0.76) | 0.53 (0.36–0.66) | 12.2 (9-15.5) |
| IL6R | 0.66 (0.49–0.80) | 0.67 (0.54–0.77) | 12.9 (10.5-15.2) |
| IL7 | 0.38 (0.17–0.56) | 0.36 (0.17–0.52) | 35.0 (29.1-40.9) |
| IL9 | 0.82 (0.66–0.93) | 0.75 (0.65–0.83) | 15.5 (11.5-19.5) |
| KDR | 0.93 (0.88–0.96) | 0.99 (0.99–0.99) | 9.8 (7.7-11.8) |
| KLK6 | 0.54 (0.37–0.67) | 0.55 (0.39–0.68) | 14.6 (12.3-16.8) |
| MAPT | 0.83 (0.72–0.90) | 0.85 (0.78–0.90) | 11.6 (9.5-13.7) |
| MDH1 | 0.56 (0.38–0.71) | 0.49 (0.32–0.63) | 33.4 (27.3-39.4) |
| MME | 0.96 (0.93–0.98) | 0.96 (0.94–0.98) | 17.7 (14-21.5) |
| MSLN | 0.97 (0.95–0.98) | 0.97 (0.96–0.98) | 8.2 (6.5-9.9) |
| NfH | 1.00 (0.99–1.00) | 0.99 (0.99–1.00) | 9.7 (7.5-12) |
| NfL | 0.93 (0.89–0.95) | 0.93 (0.89–0.95) | 10 (8.2-11.7) |
| NGF | 0.88 (0.79–0.93) | 0.92 (0.88–0.95) | 7.0 (5.7-8.4) |
| NPTX1 | 0.87 (0.77–0.92) | 0.90 (0.85–0.93) | 7.0 (5.7-8.2) |
| NPTX2 | 0.89 (0.82–0.93) | 0.90 (0.86–0.94) | 6.6 (5.5-7.6) |
| NPTXR | 0.58 (0.41–0.72) | 0.59 (0.44–0.71) | 19.1 (16.2-22) |
| NPY | 0.67 (0.50–0.79) | 0.87 (0.81–0.91) | 17.8 (13.5-22.1) |
| NRGN | 0.53 (0.35–0.67) | 0.55 (0.39–0.68) | 63.9 (55.1-72.6) |
| Oligo-SNCA | 0.40 (0.19–0.58) | 0.35 (0.16–0.52) | 68.4 (59.6-77.2) |
| PARK7 | 0.84 (0.76–0.89) | 0.78 (0.69–0.85) | 22.1 (16.5-27.6) |
| PDGFRB | 0.92 (0.86–0.96) | 0.96 (0.94–0.97) | 7.4 (6.2-8.7) |
| PDLIM5 | 0.92 (0.85–0.96) | 0.96 (0.94–0.97) | 14.5 (10.1-18.8) |
| PGF | 0.77 (0.63–0.87) | 0.81 (0.73–0.87) | 8.7 (7.1-10.3) |
| PGK1 | 0.47 (0.26–0.63) | 0.41 (0.22–0.56) | 48.5 (40.4-56.6) |
| POSTN | 0.86 (0.77–0.92) | 0.91 (0.86–0.94) | 9.4 (7.7-11.1) |
| PRDX6 | 0.63 (0.46–0.76) | 0.61 (0.46–0.72) | 34.4 (28.2-40.6) |
| PSEN1 | 0.46 (0.26–0.63) | 0.42 (0.24–0.58) | 22.1 (17.2-27.1) |
| pSNCA-129 | 0.42 (0.22–0.59) | 0.36 (0.17–0.52) | 44.1 (36.8-51.4) |
| p-tau181 | 0.93 (0.82–0.95) | 0.92 (0.88–0.95) | 12.5 (10.4-14.5) |
| p-tau217 | 0.94 (0.90–0.96) | 0.95 (0.92–0.97) | 13.5 (11.5-15.6) |
| p-tau231 | 0.93 (0.88–0.95) | 0.93 (0.89–0.95) | 12.4 (10.2-14.5) |
| pTDP43-409 | 0.69 (0.54–0.80) | 0.71 (0.60–0.80) | 15.4 (11.4-19.4) |
| PTN | 0.88 (0.79–0.93) | 0.99 (0.98–0.99) | 9.9 (7.8-12) |
| REST | 0.61 (0.44–0.76) | 0.72 (0.61–0.81) | 14.0 (10.3-17.8) |
| RUVBL2 | 0.68 (0.53–0.78) | 0.66 (0.53–0.76) | 17.4 (13.6-21.2) |
| S100A12 | 0.51 (0.31–0.68) | 0.38 (0.19–0.54) | 38.2 (30.7-45.7) |
| S100B | 0.74 (0.59–0.84) | 0.65 (0.51–0.75) | 13.7 (10.3-17.2) |
| SAA1 | 0.75 (0.57–0.90) | 0.72 (0.61–0.81) | 25.7 (18.7-32.8) |
| SFRP1 | 0.57 (0.39–0.70) | 0.62 (0.48–0.73) | 31.3 (26.3-36.3) |
| SFTPD | 0.97 (0.95–0.98) | 0.97 (0.95–0.98) | 7.6 (6.3-8.8) |
| SLIT2 | 0.73 (0.60–0.83) | 0.79 (0.69–0.85) | 7.8 (6.6-9.1) |
| SMOC1 | 0.83 (0.75–0.89) | 0.94 (0.90–0.96) | 14.0 (11.9-16.1) |
| SNAP25 | 0.61 (0.44–0.74) | 0.72 (0.60–0.80) | 3.5 (2.9-4.2) |
| SNCA | 0.42 (0.21–0.60) | 0.38 (0.19–0.54) | 36.5 (30.5-42.6) |
| SNCB | 0.78 (0.67–0.85) | 0.78 (0.69–0.85) | 7.8 (6.2-9.4) |
| SOD1 | 0.38 (0.18–0.57) | 0.33 (0.13–0.50) | 32.0 (26-38) |
| SQSTM1 | 0.57 (0.38–0.74) | 0.66 (0.53–0.76) | 19.6 (15-24.2) |
| TAFA5 | 0.86 (0.79–0.91) | 0.87 (0.82–0.92) | 11.6 (9.6-13.7) |
| TARDBP | 0.34 (0.13–0.54) ** | 0.41 (0.23–0.57) | 44.5 (37.2-51.9) |
| TEK | 0.71 (0.56–0.82) | 0.77 (0.67–0.84) | 7.2 (6-8.4) |
| TIMP3 | 0.62 (0.47–0.74) | 0.53 (0.37–0.66) | 34.5 (28.2-40.8) |
| TNF | 0.72 (0.54–0.85) | 0.77 (0.67–0.84) | 9.1 (7.1-11.1) |
| TREM1 | 0.85 (0.75–0.91) | 0.86 (0.79–0.90) | 9.9 (8.1-11.7) |
| TREM2 | 0.93 (0.88–0.95) | 0.94 (0.91–0.96) | 9.0 (7.5-10.4) |
| UBB | 0.94 (0.88–0.97) | 0.96 (0.94–0.98) | 8.8 (5.9-11.8) |
| UCHL1 | 0.79 (0.70–0.85) | 0.95 (0.92–0.96) | 4.4 (3.7-5) |
| VCAM1 | 0.77 (0.64–0.85) | 0.77 (0.68–0.85) | 7.9 (6.4-9.3) |
| VEGFA | 0.75 (0.64–0.84) | 0.81 (0.72–0.87) | 7.5 (6.3-8.6) |
| VEGFD | 0.80 (0.67–0.88) | 0.92 (0.89–0.95) | 11.0 (8.7-13.2) |
| VGF | 0.88 (0.76–0.95) | 0.94 (0.91–0.96) | 18.6 (14.1-23.1) |
| VSNL1 | 0.80 (0.69–0.88) | 0.85 (0.78–0.90) | 8.3 (6.8-9.8) |
| YWHAG | 0.27 (0.08–0.45) ** | 0.45 (0.28–0.60) | 10.4 (8.9-11.9) |
| YWHAZ | 0.13 (-0.09–0.33) ^NS^ | 0.15 (-0.06–0.34) | 11.7 (9.1-14.4) |

Test–retest reliability was evaluated for plasma biomarkers measured using NULISAseq CNS Disease Panel (Alamar Biosciences), N=92, using Spearman rho, intraclass correlation coefficient (ICC), and within-subject coefficient of variation (CV_w_). Values are presented with corresponding 95% confidence intervals (CIs) where applicable, Spearman rho and ICC both have lower bounds of 0 and upper bounds of 1. Spearman rho p < 0.001 unless otherwise indicated; **p < 0.01, ^NS^(non-significant)p < 0.05.

## **Supplementary Table 5. Association between inter-sample interval and within-subject variability of plasma biomarkers**

| Biomarker | Assay | Beta (95% CI) | Adjusted p-value |
| --- | --- | --- | --- |
| ACHE | NULISAseq | 0.16 (0.05-0.28) | 0.028* |
| AGRN | NULISAseq | 0.07 (0.01-0.13) | 0.096 |
| ANXA5 | NULISAseq | 0.31 (-0.15-0.76) | 0.353 |
| APOE | NULISAseq | 0.01 (-0.1-0.12) | 0.907 |
| APOE e4 | NULISAseq | 0.39 (-0.35-1.13) | 0.465 |
| ARSA | NULISAseq | 0.00 (-0.31-0.31) | 0.990 |
| Aβ38 | NULISAseq | 0.03 (-0.2-0.27) | 0.864 |
| Aβ40 | NULISAseq | 0.03 (-0.14-0.21) | 0.839 |
| Aβ42 | NULISAseq | 0.01 (-0.16-0.18) | 0.976 |
| Aβ42 | NULISAqpcr | 0.00 (-0.16-0.15) | 0.973 |
| Aβ42/40 | NULISAseq | 0.16 (-0.06–0.38) | 0.281 |
| BACE1 | NULISAseq | 0.03 (-0.03-0.09) | 0.470 |
| BASP1 | NULISAseq | 0.12 (0-0.24) | 0.152 |
| BDMAPT | NULISAseq | 0.02 (-0.04-0.08) | 0.652 |
| BDNF | NULISAseq | 0.12 (-0.19-0.43) | 0.606 |
| BD-p-tau181 | NULISAseq | 0.01 (-0.12-0.14) | 0.961 |
| BD-p-tau217 | NULISAseq | 0.06 (-0.04-0.16) | 0.364 |
| BD-p-tau217 | NULISAqpcr | 0.11 (-0.10–0.33) | 0.481 |
| BD-p-tau217/Aβ42 | NULISAqpcr | 0.03 (-0.17–0.22) | 0.885 |
| BD-p-tau231 | NULISAseq | 0.04 (-0.01-0.1) | 0.264 |
| CALB2 | NULISAseq | 0.10 (0-0.21) | 0.134 |
| CCL11 | NULISAseq | 0.04 (-0.03-0.11) | 0.382 |
| CCL13 | NULISAseq | 0.18 (0-0.35) | 0.141 |
| CCL17 | NULISAseq | 0.33 (-0.01-0.67) | 0.152 |
| CCL2 | NULISAseq | 0.15 (0.03-0.28) | 0.072 |
| CCL22 | NULISAseq | 0.11 (0.04-0.18) | 0.017* |
| CCL26 | NULISAseq | 0.07 (-0.04-0.19) | 0.353 |
| CCL3 | NULISAseq | 0.22 (0.08-0.35) | 0.013* |
| CCL4 | NULISAseq | 0.14 (0.02-0.27) | 0.087 |
| CD40LG | NULISAseq | 0.24 (-0.1-0.58) | 0.323 |
| CD63 | NULISAseq | 0.08 (0.02-0.14) | 0.031* |
| CHI3L1 | NULISAseq | 0.19 (0.06-0.31) | 0.019* |
| CHIT1 | NULISAseq | 0.06 (-0.17-0.29) | 0.792 |
| CNTN2 | NULISAseq | 0.19 (-0.04-0.42) | 0.247 |
| CRH | NULISAseq | 0.18 (0.09-0.28) | 0.002** |
| CRP | NULISAseq | 0.53 (0.26-0.8) | 0.002** |
| CSF2 | NULISAseq | 0.07 (-0.02-0.16) | 0.270 |
| CST3 | NULISAseq | 0.09 (0.04-0.14) | 0.009** |
| CX3CL1 | NULISAseq | 0.05 (-0.01-0.11) | 0.232 |
| CXCL1 | NULISAseq | 0.25 (-0.04-0.54) | 0.222 |
| CXCL10 | NULISAseq | 0.21 (0-0.42) | 0.134 |
| CXCL8 | NULISAseq | 0.38 (0.15-0.61) | 0.011* |
| DDC | NULISAseq | 0.29 (0.19-0.38) | 0.000*** |
| ENO2 | NULISAseq | 0.14 (-0.08-0.37) | 0.364 |
| FABP3 | NULISAseq | 0.04 (-0.06-0.15) | 0.605 |
| FCN2 | NULISAseq | -0.07 (-0.14-0.01) | 0.190 |
| FGF2 | NULISAseq | 0.32 (-0.06-0.71) | 0.241 |
| FLT1 | NULISAseq | 0.00 (-0.09-0.1) | 0.990 |
| FOLR1 | NULISAseq | 0.00 (-0.08-0.08) | 0.990 |
| GDF15 | NULISAseq | 0.00 (-0.18-0.18) | 0.990 |
| GDI1 | NULISAseq | 0.09 (-0.09-0.26) | 0.470 |
| GDNF | NULISAseq | 0.31 (0.19-0.42) | 0.000*** |
| GFAP | NULISAseq | 0.13 (0.02-0.24) | 0.072 |
| GFAP | NULISAqpcr | 0.11 (-0.01–0.23) | 0.179 |
| GOT1 | NULISAseq | 0.02 (-0.08-0.13) | 0.819 |
| HBA1 | NULISAseq | 0.16 (-0.25-0.58) | 0.605 |
| HTT | NULISAseq | 0.24 (-0.13-0.61) | 0.364 |
| ICAM1 | NULISAseq | 0.01 (-0.07-0.09) | 0.886 |
| IFNG | NULISAseq | 0.54 (0.27-0.82) | 0.002** |
| IGF1R | NULISAseq | 0.01 (-0.05-0.08) | 0.822 |
| IGFBP7 | NULISAseq | 0.04 (-0.01-0.1) | 0.272 |
| IL10 | NULISAseq | 0.14 (-0.03-0.31) | 0.243 |
| IL12p70 | NULISAseq | 0.16 (0.02-0.3) | 0.087 |
| IL13 | NULISAseq | 0.16 (0.02-0.29) | 0.087 |
| IL15 | NULISAseq | 0.02 (-0.06-0.1) | 0.760 |
| IL16 | NULISAseq | -0.04 (-0.24-0.16) | 0.832 |
| IL17A | NULISAseq | 0.18 (0.07-0.3) | 0.010* |
| IL18 | NULISAseq | 0.15 (0-0.3) | 0.134 |
| IL1B | NULISAseq | 0.16 (-0.1-0.42) | 0.382 |
| IL2 | NULISAseq | 0.21 (0.1-0.33) | 0.005** |
| IL33 | NULISAseq | 0.25 (0.11-0.39) | 0.007** |
| IL4 | NULISAseq | 0.18 (0.03-0.32) | 0.072 |
| IL5 | NULISAseq | 0.27 (0.15-0.4) | 0.001** |
| IL6 | NULISAseq | 0.23 (0.07-0.39) | 0.022 |
| IL6R | NULISAseq | 0.06 (-0.06-0.18) | 0.465 |
| IL7 | NULISAseq | 0.35 (0.06-0.64) | 0.072 |
| IL9 | NULISAseq | 0.39 (0.2-0.58) | 0.001** |
| KDR | NULISAseq | 0.01 (-0.09-0.11) | 0.934 |
| KLK6 | NULISAseq | 0.09 (-0.02-0.2) | 0.247 |
| MAPT | NULISAseq | 0.15 (0.05-0.25) | 0.022* |
| MDH1 | NULISAseq | 0.19 (-0.12-0.5) | 0.382 |
| MME | NULISAseq | 0.48 (0.32-0.64) | 0.000*** |
| MSLN | NULISAseq | 0.15 (0.07-0.23) | 0.002** |
| NfH | NULISAseq | 0.01 (-0.1-0.12) | 0.931 |
| NfL | NULISAseq | 0.12 (0.03-0.21) | 0.031* |
| NfL | NULISAqpcr | 0.17 (0.08–0.25) | 0.004 ** |
| NGF | NULISAseq | 0.05 (-0.02-0.11) | 0.353 |
| NPTX1 | NULISAseq | 0.00 (-0.06-0.07) | 0.968 |
| NPTX2 | NULISAseq | 0.01 (-0.05-0.06) | 0.915 |
| NPTXR | NULISAseq | 0.07 (-0.07-0.22) | 0.465 |
| NPY | NULISAseq | 0.05 (-0.17-0.27) | 0.825 |
| NRGN | NULISAseq | 0.29 (-0.16-0.74) | 0.364 |
| Oligo-SNCA | NULISAseq | 0.18 (-0.27-0.63) | 0.601 |
| PARK7 | NULISAseq | 0.08 (-0.2-0.35) | 0.760 |
| PDGFRB | NULISAseq | 0.03 (-0.04-0.09) | 0.576 |
| PDLIM5 | NULISAseq | 0.47 (0.28-0.66) | 0.000*** |
| PGF | NULISAseq | 0.10 (0.02-0.18) | 0.062 |
| PGK1 | NULISAseq | 0.28 (-0.14-0.69) | 0.353 |
| POSTN | NULISAseq | 0.10 (0.01-0.18) | 0.089 |
| PRDX6 | NULISAseq | 0.05 (-0.27-0.36) | 0.864 |
| PSEN1 | NULISAseq | 0.05 (-0.21-0.3) | 0.860 |
| pSNCA-129 | NULISAseq | 0.19 (-0.18-0.57) | 0.465 |
| p-tau181 | NULISAseq | 0.12 (0.02-0.22) | 0.086 |
| p-tau217 | NULISAseq | 0.08 (-0.02-0.19) | 0.247 |
| p-tau217 | Lumipulse G | 0.09 (-0.01–0.19) | 0.189 |
| p-tau231 | NULISAseq | 0.05 (-0.06-0.16) | 0.595 |
| pTDP43-409 | NULISAseq | 0.06 (-0.14-0.27) | 0.702 |
| PTN | NULISAseq | -0.04 (-0.14-0.06) | 0.606 |
| REST | NULISAseq | 0.14 (-0.05-0.32) | 0.302 |
| RUVBL2 | NULISAseq | 0.03 (-0.16-0.23) | 0.860 |
| S100A12 | NULISAseq | -0.06 (-0.43-0.32) | 0.864 |
| S100B | NULISAseq | -0.04 (-0.22-0.14) | 0.804 |
| SAA1 | NULISAseq | 0.78 (0.46-1.09) | 0.000 |
| SFRP1 | NULISAseq | 0.15 (-0.09-0.4) | 0.364 |
| SFTPD | NULISAseq | 0.02 (-0.04-0.09) | 0.674 |
| SLIT2 | NULISAseq | 0.04 (-0.02-0.1) | 0.384 |
| SMOC1 | NULISAseq | 0.11 (0.01-0.21) | 0.109 |
| SNAP25 | NULISAseq | 0.01 (-0.02-0.04) | 0.637 |
| SNCA | NULISAseq | 0.14 (-0.17-0.44) | 0.569 |
| SNCB | NULISAseq | 0.01 (-0.07-0.1) | 0.860 |
| SOD1 | NULISAseq | -0.01 (-0.32-0.3) | 0.976 |
| SQSTM1 | NULISAseq | 0.23 (-0.01-0.46) | 0.152 |
| TAFA5 | NULISAseq | 0.11 (0-0.21) | 0.125 |
| TARDBP | NULISAseq | 0.20 (-0.18-0.57) | 0.465 |
| TEK | NULISAseq | -0.01 (-0.07-0.05) | 0.840 |
| TIMP3 | NULISAseq | 0.21 (-0.11-0.53) | 0.364 |
| TNF | NULISAseq | 0.15 (0.05-0.24) | 0.015* |
| TREM1 | NULISAseq | 0.05 (-0.04-0.14) | 0.421 |
| TREM2 | NULISAseq | 0.12 (0.05-0.19) | 0.006** |
| UBB | NULISAseq | 0.27 (0.13-0.4) | 0.002** |
| UCHL1 | NULISAseq | -0.02 (-0.05-0.01) | 0.447 |
| VCAM1 | NULISAseq | 0.10 (0.03-0.17) | 0.028* |
| VEGFA | NULISAseq | 0.08 (0.03-0.14) | 0.028* |
| VEGFD | NULISAseq | -0.02 (-0.13-0.1) | 0.860 |
| VGF | NULISAseq | 0.44 (0.23-0.65) | 0.001** |
| VSNL1 | NULISAseq | 0.09 (0.01-0.16) | 0.088 |
| YWHAG | NULISAseq | 0.06 (-0.01-0.14) | 0.247 |
| YWHAZ | NULISAseq | -0.02 (-0.16-0.11) | 0.860 |

Linear regression models were used to assess whether longer time between paired samples was associated with greater within-subject variability, measured as the within-subject coefficient of variation (CV_w_) adjusting for age and sex. Effect estimates, standard errors, and p-values are reported. False discovery rate (FDR) correction was applied to account for multiple comparisons. Adjusted p-value NS (non-significant) unless other indicated; ***p< 0.001, **p < 0.01, *p < 0.05. Full assay names: Lumipulse G; Lumipulse G plasma p-tau217 (N=92), NULISAseq; NULISAseq CNS Disease Panel (N=92), NULISAqpcr; NULISAqPCR (quantitative polymerase chain reaction) AD 5-Plex assay (N=80).

## **Supplementary Table 6. Plasma biomarkers measured using the NULISAseq CNS Disease Panel, with full names and manufacturer-defined CNS panel categories (Alamar Biosciences)**

| Biomarker | Full name | CNS Panel Category |
| --- | --- | --- |
| ACHE | Acetylcholinesterase | A+T pathology |
| AGRN | Agrin | Synuclein and synaptic disorders |
| ANXA5 | Annexin A5 | Neurodegeneration |
| APOE | Apolipoprotein E | A+T pathology |
| APOE ε4 | Apolipoprotein E epsilon 4 allele | A+T pathology |
| ARSA | Arylsulfatase A | Synuclein and synaptic disorders |
| Aβ38 | Amyloid beta 38 | A+T pathology |
| Aβ40 | Amyloid beta 40 | A+T pathology |
| Aβ42 | Amyloid beta 42 | A+T pathology |
| BACE1 | Beta-secretase 1 | A+T pathology |
| BASP1 | Brain abundant membrane attached signal protein 1 | A+T pathology |
| BDMAPT | Brain-derived microtubule-associated protein tau | A+T pathology |
| BD-p-tau181 | Brain-derived phosphorylated tau 181 | A+T pathology |
| BD-p-tau217 | Brain-derived phosphorylated tau 217 | A+T pathology |
| BD-p-tau231 | Brain-derived phosphorylated tau 231 | A+T pathology |
| BDNF | Brain-derived neurotrophic factor | Synuclein and synaptic disorders |
| CALB2 | Calbindin 2 | Neurodegeneration |
| CCL11 | C-C motif chemokine ligand 11 | Inflammation |
| CCL13 | C-C motif chemokine ligand 13 | Inflammation |
| CCL17 | C-C motif chemokine ligand 17 | Inflammation |
| CCL2 | C-C motif chemokine ligand 2 | Inflammation |
| CCL22 | C-C motif chemokine ligand 22 | Inflammation |
| CCL26 | C-C motif chemokine ligand 26 | Inflammation |
| CCL3 | C-C motif chemokine ligand 3 | Inflammation |
| CCL4 | C-C motif chemokine ligand 4 | Inflammation |
| CD40LG | CD40 ligand | Inflammation |
| CD63 | Cluster of differentiation 63 | A+T pathology |
| CHI3L1 | Chitinase-3-like protein 1 | Inflammation |
| CHIT1 | Chitotriosidase 1 | Inflammation |
| CNTN2 | Contactin 2 | Neurodegeneration |
| CRH | Corticotropin-releasing hormone | Inflammation |
| CRP | C-reactive protein | Inflammation |
| CSF2 | Colony stimulating factor 2 | Inflammation |
| CST3 | Cystatin C | A+T pathology |
| CX3CL1 | C-X3-C motif chemokine ligand 1 | Inflammation |
| CXCL1 | C-X-C motif chemokine ligand 1 | Inflammation |
| CXCL10 | C-X-C motif chemokine ligand 10 | Inflammation |
| CXCL8 | C-X-C motif chemokine ligand 8 | Inflammation |
| DDC | Dopa decarboxylase | Synuclein and synaptic disorders |
| ENO2 | Enolase 2 | Neurodegeneration |
| FABP3 | Fatty acid binding protein 3 | Synuclein and synaptic disorders |
| FCN2 | Ficolin 2 | Inflammation |
| FGF2 | Fibroblast growth factor 2 | Neurodegeneration |
| FLT1 | Fms-related tyrosine kinase 1 | Vascular and metabolism |
| FOLR1 | Folate receptor 1 | Synuclein and synaptic disorders |
| GDF15 | Growth differentiation factor 15 | Inflammation |
| GDI1 | GDP dissociation inhibitor 1 | Neurodegeneration |
| GDNF | Glial cell line-derived neurotrophic factor | Neurodegeneration |
| GFAP | Glial fibrillary acidic protein | Inflammation |
| GOT1 | Glutamic-oxaloacetic transaminase 1 | Neurodegeneration |
| HBA1 | Hemoglobin subunit alpha 1 | Vascular |
| HTT | Huntingtin | Synuclein and synaptic disorders |
| ICAM1 | Intercellular adhesion molecule 1 | Inflammation |
| IFNG | Interferon gamma | Inflammation |
| IGF1R | Insulin-like growth factor 1 receptor | Inflammation |
| IGFBP7 | Insulin-like growth factor binding protein 7 | A+T pathology |
| IL10 | Interleukin 10 | Inflammation |
| IL12p70 | Interleukin 12 p70 | Inflammation |
| IL13 | Interleukin 13 | Inflammation |
| IL15 | Interleukin 15 | Inflammation |
| IL16 | Interleukin 16 | Inflammation |
| IL17A | Interleukin 17A | Inflammation |
| IL18 | Interleukin 18 | Inflammation |
| IL1B | Interleukin 1 beta | Inflammation |
| IL2 | Interleukin 2 | Inflammation |
| IL33 | Interleukin 33 | Inflammation |
| IL4 | Interleukin 4 | Inflammation |
| IL5 | Interleukin 5 | Inflammation |
| IL6 | Interleukin 6 | Inflammation |
| IL6R | Interleukin 6 receptor | Synuclein and synaptic disorders |
| IL7 | Interleukin 7 | Inflammation |
| IL9 | Interleukin 9 | Inflammation |
| KDR | Kinase insert domain receptor | Vascular and metabolism |
| KLK6 | Kallikrein related peptidase 6 | A+T pathology |
| MAPT | Microtubule associated protein tau | A+T pathology |
| MDH1 | Malate dehydrogenase 1 | Synuclein and synaptic disorders |
| MME | Membrane metalloendopeptidase | Vascular and metabolism |
| MSLN | Mesothelin | Neurodegeneration |
| NfH | Neurofilament heavy chain | Neurodegeneration |
| NfL | Neurofilament light chain | Neurodegeneration |
| NGF | Nerve growth factor | Synuclein and synaptic disorders |
| NPTX1 | Neuronal pentraxin 1 | Neurodegeneration |
| NPTX2 | Neuronal pentraxin 2 | Neurodegeneration |
| NPTXR | Neuronal pentraxin receptor | Neurodegeneration |
| NPY | Neuropeptide Y | Neurodegeneration |
| NRGN | Neurogranin | Neurodegeneration |
| Oligo-SNCA | Oligomeric alpha-synuclein | Synuclein and synaptic disorders |
| PARK7 | Parkinsonism associated deglycase | Synuclein and synaptic disorders |
| PDGFRB | Platelet-derived growth factor receptor beta | Vascular and metabolism |
| PDLIM5 | PDZ and LIM domain protein 5 | Neurodegeneration |
| PGF | Placental growth factor | Vascular |
| PGK1 | Phosphoglycerate kinase 1 | Vascular and metabolism |
| POSTN | Periostin | Vascular and metabolism |
| PRDX6 | Peroxiredoxin 6 | Inflammation |
| PSEN1 | Presenilin 1 | A+T pathology |
| pSNCA-129 | Phosphorylated alpha-synuclein serine 129 | Synuclein and synaptic disorders |
| p-tau181 | Phosphorylated tau 181 | A+T pathology |
| p-tau217 | Phosphorylated tau 217 | A+T pathology |
| p-tau231 | Phosphorylated tau 231 | A+T pathology |
| pTDP43-409 | Phosphorylated TDP-43 serine 409/410 | Synuclein and synaptic disorders |
| PTN | Pleiotrophin | A+T pathology |
| REST | RE1-silencing transcription factor | Neurodegeneration |
| RUVBL2 | RuvB-like AAA ATPase 2 | Inflammation |
| S100A12 | S100 calcium-binding protein A12 | Inflammation |
| S100B | S100 calcium-binding protein B | Inflammation |
| SAA1 | Serum amyloid A1 | Vascular |
| SFRP1 | Secreted frizzled related protein 1 | A+T pathology |
| SFTPD | Surfactant protein D | Inflammation |
| SLIT2 | Slit guidance ligand 2 | Inflammation |
| SMOC1 | SPARC related modular calcium binding 1 | Neurodegeneration |
| SNAP25 | Synaptosomal-associated protein 25 | Neurodegeneration |
| SNCA | Alpha-synuclein | Synuclein and synaptic disorders |
| SNCB | Beta-synuclein | Synuclein and synaptic disorders |
| SOD1 | Superoxide dismutase 1 | Synuclein and synaptic disorders |
| SQSTM1 | Sequestosome 1 | Neurodegeneration |
| TAFA5 | TAFA chemokine-like family member 5 | Inflammation |
| TARDBP | TAR DNA-binding protein | Synuclein and synaptic disorders |
| TEK | TEK receptor tyrosine kinase | Inflammation |
| TIMP3 | Tissue inhibitor of metalloproteinases 3 | Inflammation |
| TNF | Tumor necrosis factor | Inflammation |
| TREM1 | Triggering receptor expressed on myeloid cells 1 | Inflammation |
| TREM2 | Triggering receptor expressed on myeloid cells 2 | Inflammation |
| UBB | Ubiquitin B | Neurodegeneration |
| UCHL1 | Ubiquitin C-terminal hydrolase L1 | Synuclein and synaptic disorders |
| VCAM1 | Vascular cell adhesion molecule 1 | Inflammation |
| VEGFA | Vascular endothelial growth factor A | Vascular |
| VEGFD | Vascular endothelial growth factor D | Vascular |
| VGF | VGF nerve growth factor inducible | Vascular |
| VSNL1 | Visinin-like protein 1 | Neurodegeneration |
| YWHAG | Tyrosine 3-monooxygenase activation protein gamma | Neurodegeneration |
| YWHAZ | Tyrosine 3-monooxygenase activation protein zeta | Neurodegeneration |

**Supplementary Figure 1: Test-retest reliability metrics for plasma biomarkers quantified using the NULISAseq CNS Disease Panel (Alamar Biosciences) ordered by manufacturer defined panel category.** Biomarkers were ranked according to their performance for each reliability metric. Darker colours for Spearman rho and intraclass correlation coefficient (ICC) indicate higher correlations and test-retest reliability, with lighter colour for within-subject coefficient of variation (CV_w_) indicating lower within-subject variability. Spearman rho and ICC both have lower bounds of 0 and upper bounds of 1.

a

b

**Supplementary Figure 2. Cross-assay correlation of plasma p-tau217 and BD-p-tau217.** (a) Correlation of mean p-tau217 measured on both the NULISAseq CNS Disease Panel (NPQ) and Lumipulse G (pg/mL) (b) Correlation of mean BD-p-tau217 measured on both the NULISAseq CNS Disease Panel (NPQ) and NULISAqpcr AD 5-plex assay (pg/mL).

**Supplementary Figure 3. Plasma biomarkers, measured on the NULISAseq CNS Disease Panel, association between inter-sample interval and within-subject variability (CV_w_) following false discovery rate (FDR) correction.** Analyses were adjusted for age and sex. Each point represents an individual biomarker effect estimate and error bars represent corresponding 95% confidence intervals (CIs). Adjusted p-value NS (non-significant) unless other indicated; ***< 0.001, **p < 0.01, *p < 0.05.
